# 

**Published:** 1899-06

**Authors:** 


### Communicated.

EDITOR "AMERICAN JOURNAL OF DENTAL SCIENCE:"

NEWARK, N. J., May 25th, 1899.

The Sixteeth Annual Session of the National Association of Dental Examiners will be held at Niagara Falls commencing at 10 o'clock a. m., Friday July 28th, and continuing the 29th, 31st and Aug. 1st, 1899. The Hotel and Hall where the sessions will be held, will be given in the July number of this Journal. It is earnestly desired that delegate members from every state will be represented this year.

CHARLES A. MEEKER, D. D. S., Sec'ty.

### NATIONAL DENTAL ASSOCIATION

The Annual Meeting of the National Dental Association will be held in the ball-room of the International Hotel, at Niagara Falls, August 1, 2, 3 and 4, 1899.

A railroad rate of one and one-third fare on the certificate plan will be obtained. Also reduced rates on C. & B. and Northern Transportation S. S. Lines. It is suggested that members living at a considerable distance organize parties, and thereby be enabled to secure lower rates from railroad companies.

Following is a list of hotels:

| | | | |
|---|---|---|---|
| Cataract House, . . . | $3.00 to | $4.00 | per day. |
| International Hotel, . . | 3.00 to | 4.00 | " |
| Kaltenback Hotel, . . | | 3.00 | " |
| Imperial Hotel. . . . | 2.50 to | 4.00 | " |
| Columbia Hotel, . . . | 1.50 to | 2.00 | " |
| Temperance House, . . | 1.50 to | 2.00 | " |
| Niagara Falls House, . . | | 2.00 | " |
| Niagara House, . . . | | 2.00 | " |

Dr. M. O. Cooley, of Niagara Falls, N. Y., will engage rooms and answer any questions regarding local arrangements for the meeting. Definite meeting places for sections will be announced later.

It is the wish of the officers of the Association that members make special efforts to be present at section meetings, on account of the unusual number of valuable papers which must first be passed upon by the section to which they properly belong.

The following is the preliminary program:

Porcelain Enamel Inlays, Dr. N. S. Jenkins, Dresden; Orthodontia, (Illustrated), Dr. Edward H. Angle, St. Louis; The Absolute Efficiency of the Controllers of the Market for Dental Cataphoresis, Dr. W. A. Price, Cleveland; Dental Electricity, Dr. L. E. Custer, Dayton; The Practical Side of It, Dr. S. S. Stowell, Pittsfield; A Bastard Profession, Dr. E. P. Beadles, Danville; Surgical Operations in Early Infancy for Palatal Defects, Dr. Truman W. Brophy, Chicago; Cements, Dr. E. K. Wedelstaedt, Minneapolis; The Reflexes of the Three Lower Molars, Dr. James Truman, Philadelphia; Operative Dentistry, Dr. J. N. Crouse, Chicago; Gomphosis, Dr. B. H. Catching, Atlanta; Prognathism. Extraction and Delay versus Expansion and Early Attention. (Illustrated), Dr. R. Ottolengui, New York; Some Phases of the Cement Question, Dr. W. V. B. Ames, Chicago; A Study of Hare-Lip and Cleft Palate, (Illustrated), Dr. Thomas Fillebrown, Boston; Dies and Counter-Dies, Dr. Robert H. Nones, Philadelphia; Phyorrhœa Alveolaris, Dr. M. L. Rhein, New York; Constitutional Deterioration the Cause of Dental Caries, Dr. Harvey, Battle Creek; Oral Affections in Secondary Syphilis, Dr. W. C. Barrett, Buffalo; The Physiological Relation of the Adult Tooth-Pulp to the Economy, Dr. C. L. Hungerford, Kansas City; Etiology of Gnathic Abnormalities, Dr. A. H. Thompson, Topeka; The Dental Profession in Charity; an Experiment in Chicago, Dr. Carl Theodore Gramm, Chicago; Some New Points in the Anatomy of the Face and Jaws, Dr. M. H. Cryer, Philadelphia; An Important Paper, by Dr. J. Leon Williams, of London.

SUBJECTS TO BE ANNOUNCED.

Dr. W. Geo. Beers, Montreal; Dr. H. L. Ambler, Cleveland; Dr. Joseph Head, Philadelphia; Dr. John S. Marshall, Chicago; Dr. A. H. Peck, Chicago; Dr. R. H. Hofheinz, Rochester; Dr. G. V. I. Brown, Milwaukee; Dr. H. H. Johnson, Macon; Dr. C. Edmund Kells, New Orleans; Dr. L. M. Cowardin, Richmond; Dr. L. L. Dunbar, San Francisco; Dr. G. V. Black, Chicago; Dr. W. H. Whistler, Cleveland; Dr. A. W. Harlan, Chicago; Dr. C. N. Johnson, Chicago; Dr. H. J. Goslee, Chicago; Dr. F. W. Low, Buffalo; Dr. T. P. Hinman, Atlanta; Dr. B. Holly Smith, Baltimore; Dr. M. C. Smith, Lynn; Dr. Edward C. Kirk, Philadelphia; Dr. W. Ernest Walker, Pass Christian.

A revised program, with reports from chaiman of sections, will be issued later. Prominent members of the profession from abroad have been invited to be present.

The names of the gentlemen who have promised to present papers is a sufficient guarantee of the high character of work which will be done at this meeting. The minor details will be carefully looked after, and all unnecessary and irrelevant matter eliminated, so that the business of the Association may be transacted in a prompt and expeditious manner. It is hoped that the various state Societies will send full delegations, and that all members of the Association, and reputable dentists in this country and Canada who are not members, will show their interest in and loyalty to the National Association by attending this meeting.

H. J. BURKHART, President.

EMMA EAMES CHASE, Cor. Secretary.

J. N. CROUSE, Chairman Ex. Committee.

### To Readers and Correspondents.

Communications intended for publication, and exchange journals and papers, should be addressed to the Editor of The American Journal of Dental Science, No. 845 N. Eutaw St. Baltimore, Md. All letters relating to the business of the Journal, or containing cash remittances, to be directed to Snowden & Cowman Mfg. Co., 9 W. Fayette Street, Baltimore, Md. Articles for publication should be received by the Editor at least two weeks previous to the first of the month on which the number in which it is designed for them to appear is issued.

☞ The American Journal of Dental Science will contain fifty pages of reading matter in each number, making a volume of about Six Hundred pages,

EXCLUSIVE OF ADVERTISEMENTS.

